# Decoding Malignant Lung Ground Glass Opacities: A Radiologic–Pathologic Study From Pure to Semi‐Solid Lesions

**DOI:** 10.1111/1759-7714.70384

**Published:** 2026-08-28

**Authors:** Alessandro Bonis, Giulia Pagliarini, Vincenzo Verzeletti, Sara Cecchinato, Anna Michielin, Serena Zanardo, Marco De Silvestro, Luigi Lione, Chiara Giraudo, Eleonora Faccioli, Giorgio Cannone, Marco Mammana, Alessandro Rebusso, Giovanni Maria Comacchio, Marco Schiavon, Andrea Dell'Amore

**Affiliations:** ^1^ Thoracic Surgery Unit, Department of Cardiac, Thoracic, Vascular Sciences and Public Health—DCTV University of Padova Padova Italy; ^2^ Unit of Advanced Clinical and Translational Imaging, Department of Cardiac, Thoracic, Vascular Sciences and Public Health—DCTV University of Padova Padova Italy

**Keywords:** adenocarcinoma, ground‐glass opacities, pathological variables, resectable NSCLC

## Abstract

**Purpose:**

Ground‐glass opacities (GGOs) are increasingly detected due to widespread low‐dose CT screening for lung cancer. Among them, malignant GGOs (mGGOs) are a clinical challenge. This study aimed to compare the radiological and anatomopathological characteristics of pure and semi‐solid mGGOs and to identify radiological predictive factors for consolidation.

**Methods:**

A retrospective study was conducted on patients resected due to mGGOs between 2016 and 2023. Patients were classified based on whether initially pure mGGOs remained pure or developed a solid component before surgery. Radiological, pathological, and molecular characteristics were analyzed.

**Results:**

Of 135 patients, 61 (46%) mGGOs became partially solid during surveillance. Semi‐solid mGGOs had a higher acinar component (*p* = 0.038) and larger pathological diameter (20 vs. 18 mm, *p* = 0.047), while pure mGGOs had a higher lepidic percentage (20% vs. 10%, *p* = 0.026). STAS and LVI were observed in 25% and 15% of cases. Logistic regression showed pleural contact was protective against consolidation (*p* = 0.033), while arterial vascularization was predictive of consolidation (*p* = 0.028). Five‐year survival was comparable and satisfactory for both groups (81.7% for pures and 85.5% for semi‐solids). Overall, LVI but no STAS was cause of a lower survival expectancy in the entire sample, regardless of consolidation (*p* = 0.004).

**Conclusions:**

Both pure and semi‐solid mGGOs showed precocious pathological and molecular features. Surgical intervention with curative intent, even in pure GGOs, is accompanied by long expected survivals. Vascular features on imaging may help inform surgical decision‐making, whereas pathological LVI may have prognostic significance in patients with mGGOs.

## Graphical abstract

In our study, among 135 completely resected pure or partially solid malignant GGOs, the presence of a feeding arterial vessel is an independent predictor to consolidation. Pathological variables are reported as early as this stage and LVI+ mGGOs present a significant lower disease‐free survival, regardless of their consolidation.

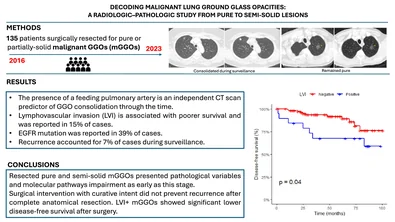

## 1 Introduction

According to GLOBOCAN statistics, lung cancer remains one of the most relevant oncological causes of death worldwide, and it is generally diagnosed in a locally advanced or advanced stage [1]. Novel screening campaigns are providing growing evidence that detecting early‐stage nonsmall cell lung cancers (NSCLC) is feasible, once having set particular parameters to screen populations at risk [2, 3, 4, 5]. The diffusion of screening programs has expanded the upfront‐resectable proportion of NSCLCs, with a significant improvement in survival. Meanwhile, by the adoption of low‐dose chest scan protocols, screenings also upwarded the detection of lung ground‐glass opacities (GGOs) [6]. The management of these lesions is challenging for at least two reasons: (1) multiple, synchronous GGOs can be detected in a single scan, but their significance is not necessarily malignant; (2) not all malignant lesions have a solid portion, which is usually a feature suggestive of invasive behavior [7]. Therefore, there is an ongoing debate about the appropriate surgical approach to these lesions, especially when a biopsy is not feasible. Thus, the aim of this study was to describe differences in radiological or anatomopathological features between malignant GGOs (mGGOs) which remained pure during surveillance and those which progressively became semi‐solid.

## 2 Materials and Methods

### 2.1 Study Design

Patients with mGGO referring to our tertiary center for lobectomy or segmentectomy between January 1, 2016, and June 30, 2023, were enrolled in this single‐center retrospective study.

This study was designed and conducted in accordance with the Declaration of Helsinki, and all patients gave written informed consent as in routine clinical practice including the consent to use their data/information for research purposes.

The following inclusion criteria were applied:

- Patients aged > 18 years who gave free written informed consent to participate;
- Patients with GGO lesions at imaging, malignant at the pathological examination;
- Patients treated with radical anatomical resections (lobectomies and segmentectomies) and hilar and mediastinal radical lymphadenectomy.

Patients who did not meet these criteria were excluded. Due to the retrospective structure of the study and its aim, we did not select cases in accordance with a particular schedule of GGO surveillance before surgery. Their pure appearance at the first outpatient evaluation and their pathological confirmation as malignant after resection were the only necessary criteria to be accepted for the study.

### 2.2 Data Retrieval and Patient Management

For each patient, demographics (age and sex), status (alive/dead), smoking habit, comorbidities, clinical staging (TNM), eventual recurrences, and the surgical report were recorded. The interval of time (days) between the first radiological detection of the lesion and the subsequent surgery was reported.

Surveillance after surgery was scheduled in accordance with the current guidelines with enhanced chest CT scans every 3 months for the first 2 years, twice a year for the following 2 years, and then yearly. In addition to the CT scan, the patient underwent the same timeline an upper abdomen ultrasound. In case of a radiological suspect of recurrence, the tumor board was promptly involved to plan further investigations or treatments. Recurrence was defined as locoregional (surgical margin, bronchial stump, ipsilateral lung, ipsilateral hilar, or mediastinal lymph nodes) or distant (any other site of recurrence).

### 2.3 Radiological Assessment

Patients with GGOs were referred to our outpatient clinic in which surgeons made the first selection according to the first chest CT scan available. One radiologist with 15 years of experience in thoracic imaging assessed all CT scans and performed all measurements described below. Because the imaging assessment was based on features typically evaluated in routine clinical practice (e.g., size, shape), a second rater was not involved to assess repeatability. Only initially pure mGGOs were considered for this study while solid and semi‐solid ones were excluded. A pure mGGO was defined as any opacity showing a hazy and increased density with 0% of solid component (Consolidation‐to‐Tumor Ratio—CTR0) measured at the first outpatient evaluation. A second screening was conducted at the latest CT scan performed before surgery, assessing whether a mGGO remained pure (CTR0) or became semisolid, in case of solid component with a CTR > 0%. In addition, pure mGGOs had to clearly show vessels and airways structures with no disruptions to be included [8].

**FIGURE 1:**
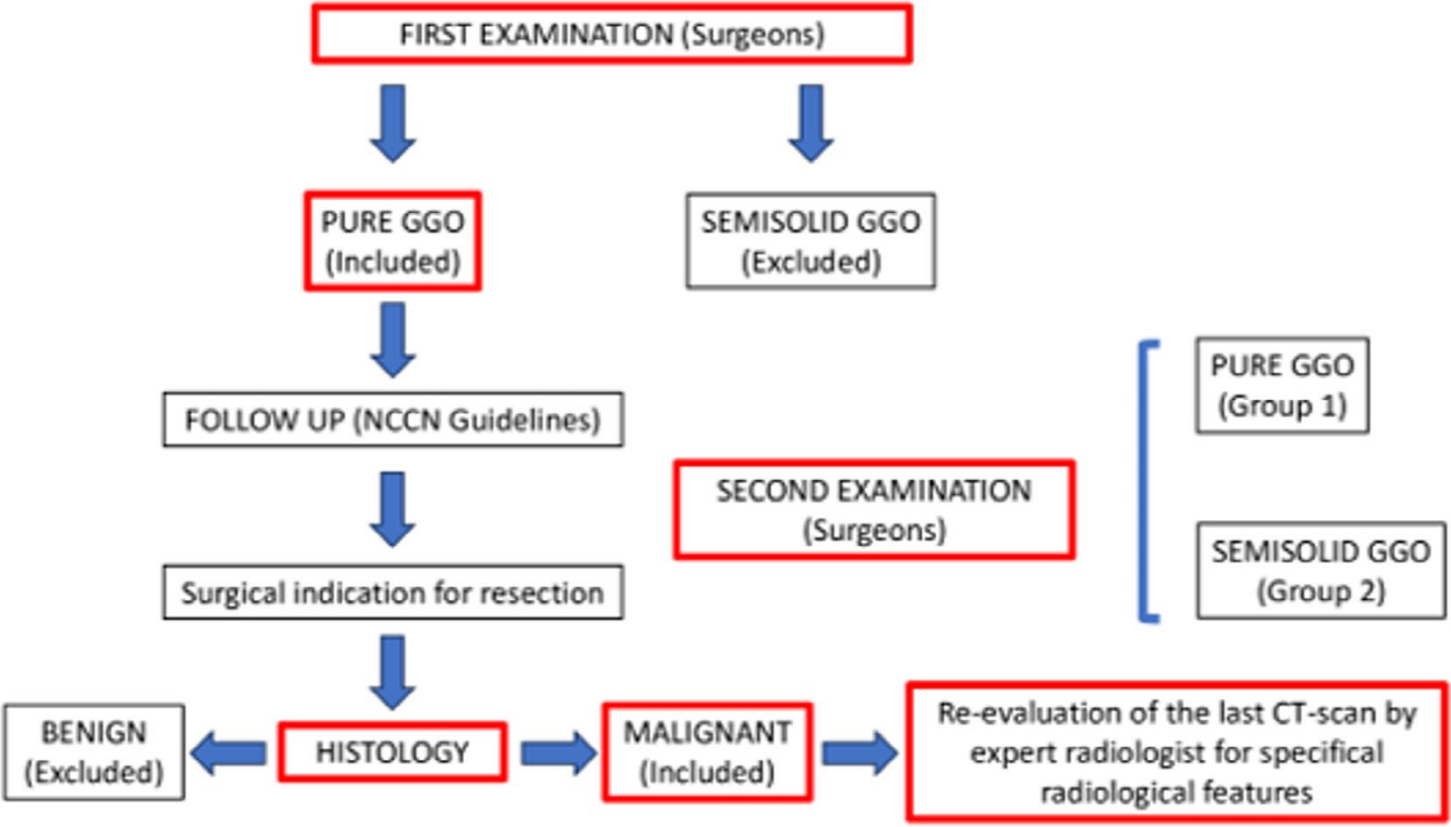
Final study design.

**FIGURE 2:**
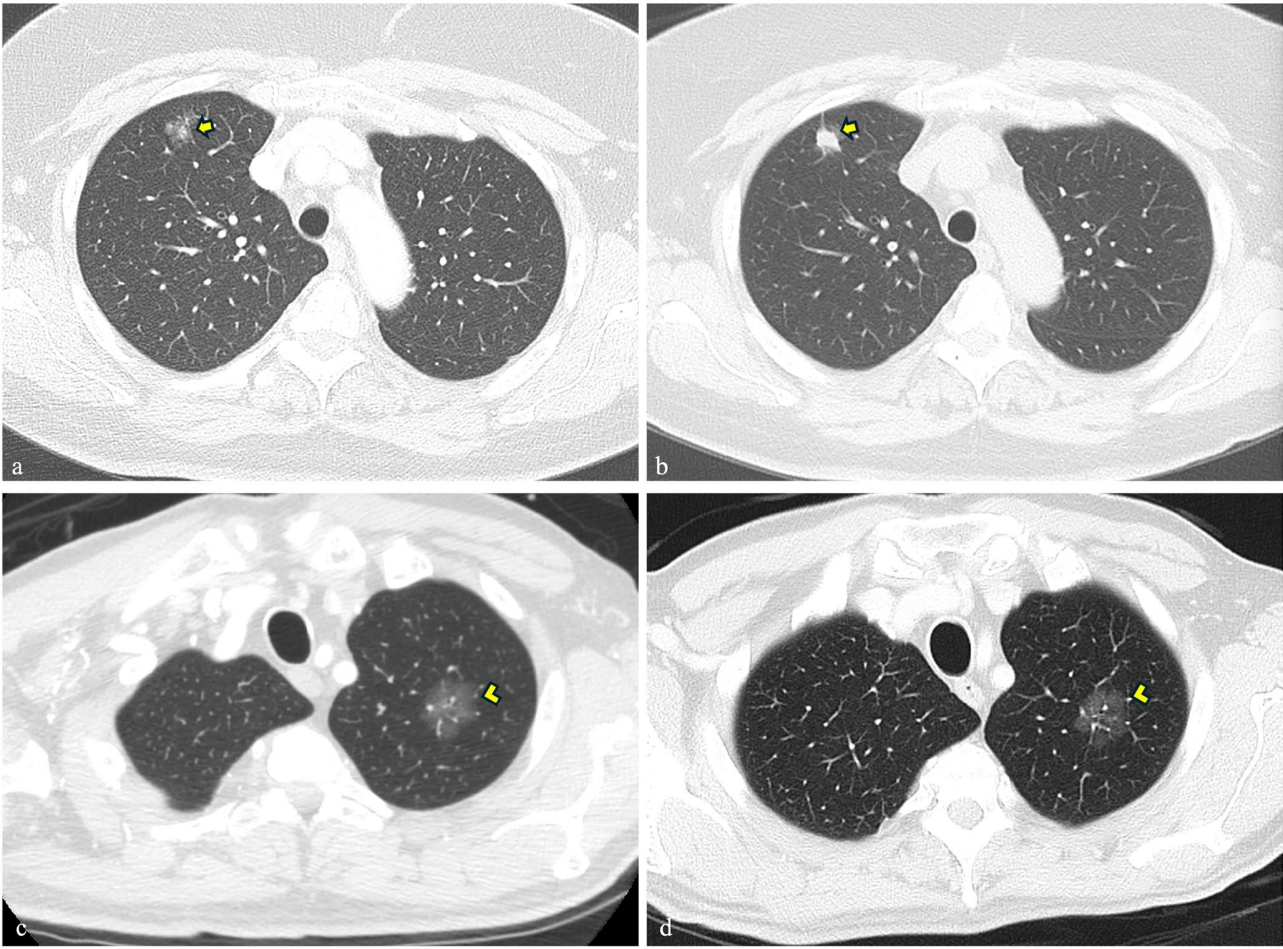
(a) Axial computed tomography of a 65‐year‐old woman with a ground glass nodule in the right upper lobe (yellow arrow) that at follow‐up 2 years later underwent consolidation (yellow arrow in b). (c) Axial computed tomography of a 73‐year‐old man with a ground glass nodule in the left upper lobe (yellow arrowhead) that at follow‐up 1 year later remained stable without any sign of consolidation (yellow arrowhead in d).

Follow‐up was scheduled in accordance with the NCCN current guidelines until evidence of surgical indication. Once resected, pure or semisolid GGOs with neoplastic diagnosis (malignant GGOs—mGGOs) were retrospectively evaluated by an expert radiologist (C.G.) to assess initial radiological features of the opacities (Figures 1 and 2).

For each mGGO the following characteristics were recorded at the initial radiologic assessment, when opacities were all pure: site, size (maximum diameter), volume (cm^3^), pleural contact, shape (round, yes/no), margins (spiculated, yes/no), bubble‐like lucency (yes/no), bronchus sign (yes/no), density (Hu) of the lesion and of the lung parenchyma. In case of contrast enhanced CT, the density in the venous phase (Hu) as well as the arterial (aka feeding vessel sign, pulmonary artery branch leading to a pulmonary nodule or opacity) and venous vascularization were assessed [9, 10].

### 2.4 Anatomopathological Assessment

Pathological data related to preoperative biopsy investigations (to record any preoperative diagnosis) and analysis of resected specimens were obtained. In particular, histotype and other histologic features of the lesion, such as growth pattern, grading, pathologic TNM staging, tumor infiltrating lymphocytes (TILs) (< 10%, between 10% and 30%, > 30%), and tumor distance from resection margins were considered. STAS (Spread Through Air Spaces) was evaluated according to the defined criteria. Within the average microscopic range ×200, STAS was further classified as low/limited STAS (1 to 4 tumor cell clusters in tumors with micropapillary or solid STAS, or 1 to 4 tumor cells in single‐cell predominant STAS) and high/extended STAS (≥ 5 clusters of tumor cells in tumors with micropapillary or solid STAS, or ≥ 5 tumor cells in single‐cell predominant STAS). Vascular infiltration was precisely defined as the evidence on the embedded specimen of invasion of the lumen of a muscular artery or vein, whether located within the tumor or close to it. Molecular investigations related to the search for alterations in EGFR, K‐RAS, PD‐L1, and ALK were also assessed. We reported data on the number of lymph node stations removed according to the IASLC classification (N1: lymph node stations from 10 to 14; N2: lymph node stations from 2 to 9) and the total number of lymph nodes in them.

### 2.5 Statistical Analysis

Continuous variables were described using median and interquartile range, and absolute number and percentage frequency were reported for categorical variables. Statistical comparisons between groups were made using the Wilcoxon rank sum test, Pearson *χ* ^2^ test, and Fisher's exact test where appropriate. The association between consolidation and radiological features was investigated with a logistic regression, after checking for collinearity confounding. Survival analysis for Overall Survival (OS) was conducted using the Kaplan–Meier estimator. Survival curves were compared using the Log‐rank test. The association between pathological features and survival was evaluated using Cox proportional hazards regression, from which hazard ratios (HRs) and 95% confidence intervals (CIs) were estimated. Before using this model, proportional hazards assumptions were separately checked using Schoenfeld residuals. The level of statistical significance was set at *p* ≤ 0.05 (Jamovi Software v2.6.44 and R Statistical Software).

## 3 Results

Overall, 135 patients divided into 69 males (51%) and 66 women (49%) with a median age of 69 years (IQR 64–73) met the inclusion criteria and were further analyzed.

According to smoking habits, 20 patients (15%) were previous smokers, 68 (50%) current smokers, and 47 (35%) nonsmokers.

In terms of localization, most of the lesions were in the right upper lobe (56 patients, 41%) followed by the upper left lobe (35 patients, 26%). Four patients had multiple lesions, two distributed in the right upper and lower lobe, and one each in the right lower lobe, middle lobe, and right upper lobe and middle lobe, respectively.

During the timeline, 38 segmentectomies (28%) and 97 lobectomies (72%) were performed. The median time interval between the diagnosis and surgery was 12 months (IQR 5–20) (Table 1).

**TABLE 1:** Characteristics of the examined population.

Clinical features	Median (IQR) or *n* (%)
Gender M/F	69 (51)/66 (49)
Age (years)	69 (64–73)
Interval between diagnosis and surgery (months)	12 (5–20)
Smoking habits	
Active^a^	68 (50)
Former	20 (15)
Never	47 (35)
Site of mGGO	
RLL	22 (16)
LLL	14 (10)
ML	4 (3)
RUL	56 (41)
LUL	35 (26)
Combined site	4 (3)
Surgical procedure	
Segmentectomy	38 (28)
Lobectomy	97 (72)

*Note:* Data are presented as median (Interquartile Range) or number (%).

Abbreviations: LLL: left lower lobe, LUL: left upper lobe, ML: middle lobe, RLL: right lower lobe, RUL: right upper lobe.

^a^ Active smokers quit between 1 and 1.5 months before surgery. Combined site designs four cases in which CT scan revealed more than a single GGO on the lung parenchyma.

### 3.1 Radiological Features

The median maximum diameter of the mGGOs was 23 mm (IQR 18–28 mm) with a median calculated volume of 4 cm^3^ (IQR 3–8). According to their aspect, 74 lesions were round‐shaped (55%), while 61 showed spiculated margins (45%). Furthermore, pleural contact was identified in 120 patients (89%). Almos half of cases (*n* = 62, 46%) showed arterial vascularization, while the bronchus sign was more commonly reported (*n* = 84, 62%). Other radiological details are summarized in Table 2.

**TABLE 2:** Radiological features of the examined population.

Radiological features	Median (IQR) or *n* (%)
Maximum diameter (mm)	23 (18–28)
Round	74 (55)
Spiculated	61 (45)
Pleural contact	120 (89)
Arterial vascularization	62 (46)
Venous vascularization	89 (59)
Bubble‐like lucency	30 (22)
Bronchus sign	84 (62)
mGGO basal (Hu)	−297 (−404, −162)
mGGO portal (Hu)	−263 (−362, −136)
Lung parenchyma value (Hu)	−884 (−905, −858)
Difference of CT value^a^	636 (491–740)
GGO volume (cm^3^)	4 (3–8)

*Note:* Data are presented as median (Interquartile Range) on number (%).

Abbreviations: CT: computed tomography, GGO: ground glass opacity, UH: hounsfield unit.

^a^ Difference of CT value is measured as the difference between the lung parenchymal HU value to the maximum GGO's HU value.

### 3.2 Anatomopathological Features

Adenocarcinoma occurred in 131 patients (97%). The remaining cases were identified as minimally invasive adenocarcinoma and squamous cell carcinoma (*n* = 4, 3%).

According to the anatomopathological report, subjects were addressed as T1a for 28 patients (21%), T1b for 30 patients (22%), T1c for 6 patients (4.4%), T2a for 63 patients (46%), T3 for 5 patients (3.7%), T4 for 2 patients (1.5%), and Tis for 1 patient (0.7%).

About N parameters, 133 patients (97%) were classified as N0, while one patient (0.7%) was classified as N1 and another one (0.7%) as N2.

When growth patterns are considered, the median for the lepidic pattern was 15% (interquartile range (IQR) 5–30), whereas the median for the acinar pattern was 80% (IQR 60–95).

The maximum anatomopathological diameter of the lesion has a median value of 18 mm (IQR 12–23). Furthermore, a median stapler‐to‐tumor distance of 25 mm (IQR 15–40) was achieved by surgeons, with a median margin‐to‐size ratio of 1.20 (IQR 0.67–2.50).

As concerns microscopical variables, STAS was reported as positive in 33 cases (25%) and LVI in 19 (15%). GGOs mostly resulted as moderately differentiated tumors (*n* = 108, 82%) and more than 40% of cases showed almost 10% of TILs in the specimen.

Immunohistochemical and genomic analysis of the tumor revealed a median value of Ki67% of 10% (IQR 5–20), ALK positive in two cases (1.6%), EGFR mutated in 47 cases (39%); KRAS wild type in 29 cases (78%), mutated to exon 2 in 8 cases (22%), and unknown in 98 cases (Table 3).

**TABLE 3:** Pathological characteristics of the entire sample.

Anatomopathological features	Median (IQR) or *n* (%)
Histotype	
ADK	131 (97)
MIA	2 (1.5)
SCC	2 (1.5)
Parameter T (pathological)	
In situ	1 (0.7)
1a	28 (21)
1b	30 (22)
1c	6 (4.4)
2a	63 (46)
Others	7 (5.3)
Parameter N (pathological)	
N0	133 (99)
N1 (station 11)	1 (0.7)
N2 (station 7)	1 (0.7)
No. of lymph nodes harvested in N1	8 (5–11)
No. of lymph nodes harvested in N2	8 (5–13)
No. of lymph node stations harvested	7 (5–8)
Tumor growth pattern	
Lepidic %	15 (5–30)
Acinar %	80 (60–95)
Surgical margins (mm)	25 (15–40)
Maximum tumor diameter (mm)	18 (12–23)
Grading	
G 1	14 (11)
G 2	108 (82)
G 3	10 (7)
Vascular invasion (LVI)	
Absent	112 (85)
Present	19 (15)
Spread through air spaces (STAS)	
Positive STAS	33 (25)
Negative STAS	93 (75)
Lymphocytic infiltration	
< 10%	72 (53)
10%–30%	45 (33)
> 30%	13 (9.6)
KI67%	10 (5–20)
PD‐L1 (> 1%)	29 (25%)
ALK	2 (1.6)
EGFR	47 (39)
KRAS Ex.2	8 (22)

*Note:* Data are presented as median (interquartile range) or number (%).

Abbreviations: ADK: adenocarcinoma, ALK: anaplastic lymphoma kinase, EGFR: epithelial growth factor receptor, KRAS: rat sarcoma kinase, MIA: minimally invasive adenocarcinoma; SCC: squamous cell carcinoma. Ki67% is the tumor proliferation index.

The median follow‐up lasted 47 months (IQR 24–70). Of the 135 cases examined, 10 (7.5%) developed recurrence within the considered surveillance time. Specifically, seven had ipsilateral pulmonary recurrence, two had nodal recurrence (one case of N1 and one case of N2), and in one case there was both ipsilateral pulmonary failure and distant recurrence (central nervous system) (Table 4).

**TABLE 4:** Recurrence rate and sites of relapse.

Characteristics of follow‐up and recurrence	Median (IQR) or *n* (%)
Recurrence	
Yes	10 (7)
No	124 (93)
Site of recurrence	
Ipsilateral lung	7 (70)
Ipsilateral lymph nodes	2 (20)
Only distant	0 (0)
Distant + Ipsilateral lung	1 (10)
Duration of follow up (months)	47 (24–70)

*Note:* Data are presented as median (interquartile range) or number (%).

### 3.3 Comparison Between Semi‐Solid and Pure mGGOs

The anatomopathological investigation highlighted some differences between pure and semi‐solid mGGOs. First, almost half of initial pure mGGOs developed a solid component during the radiological presurgical surveillance (61 cases, 46%) while 73 cases (54%) remained pure. Between these two groups several characteristics were investigated, and a few differences were observed and are detailed below. Concerning histopathological features, the lepidic component (LEP%) was unsurprisingly twofold higher in the pure group compared to consolidated (20% vs. 10%, *p* = 0.026). Otherwise, the acinar component (ACI%) was significantly more represented in the consolidated group (*p* = 0.038). Concerning tumor size, the maximum pathological tumor diameter was significantly larger in the consolidated group (20 vs. 18 mm, *p* = 0.047). No significant differences were observed between the groups in terms of tumor stage (pT1 vs. pT2‐pT4), LVI, STAS, Ki67, PD‐L1 expression, or EGFR/KRAS mutations (all with *p*‐values > 0.05).

Radiological analysis revealed that the Relative Attenuation (normal lung parenchymal HU/maximal consolidated HU) on CT was significantly higher in the pure mGGO (median of 4 vs. 2, *p* = 0.025). Further details are reported in Table 5.

**TABLE 5:** Comparative analysis between GGOs which remained as pure at surgical resection and those which consolidated due to preoperative surveillance.

Characteristic	*N*	Semi‐solid mGGO *N* = 61 (46%)	Pure mGGO *N* = 73 (54%)	*p*
Interval between diagnosis and surgery (months)	134	15 (5–23)	10 (5–17)	0.29
pT stage	134			0.89
T1		26 (43%)	32 (44%)	
T2–T4		35 (57%)	41 (56%)	
Harvested nodal stations (no.)	134	7 (5, 8)	7 (5, 8)	0.67
Lepidic %	128	10 (0, 24)	20 (5, 50)	0.026
Acinar %	128	80 (70, 95)	80 (40, 90)	0.038
Type	134			0.75
ADK		60 (98%)	70 (96%)	
MIA; AIS		0 (0%)	2 (2.7%)	
SCC		1 (1.6%)	1 (1.4%)	
STAS	129			0.56
Absent		41 (69%)	55 (79%)	
Limited		17 (29%)	14 (20%)	
Extensive		1 (1.7%)	1 (1.4%)	
LVI	130	12 (20%)	7 (10%)	0.11
Infiltrating lymphocytes	129			0.58
< 10%		34 (58%)	37 (53%)	
11%–30%		18 (31%)	27 (39%)	
> 30%		7 (12%)	6 (8.6%)	
Surgical margin (mm)	115	25 (15, 36)	30 (14, 40)	0.71
Procedure	134			0.38
Segmentectomy		15 (25%)	23 (32%)	
Lobectomy		46 (75%)	50 (68%)	
Ki67 (%)	111	0.11 (0.05, 0.20)	0.10 (0.05, 0.15)	0.41
PD‐L1 (> 1%)	118	14 (26%)	15 (23%)	0.68
ALK	122	2 (3.6%)	0 (0%)	0.21
EGFR	121	21 (38%)	26 (39%)	0.89
KRAS (Ex. 2)	37	4 (22%)	4 (21%)	> 0.99
Pathological diameter (mm)	127	20 (14, 26)	18 (12, 21)	0.047
Radiological diameter (mm)	131	25 (19, 30)	23 (18, 28)	0.22
PD‐RD difference (mm)	126	4 (1, 10)	5 (2, 10)	0.43
Anatomopathological RATIO	109	1.04 (0.59, 2.11)	1.42 (0.71, 2.72)	0.24
Radiological findings				
Round	134	32 (52%)	42 (58%)	0.56
Spiculated	134	31 (51%)	32 (44%)	0.42
Pleural contact	134	57 (93%)	62 (85%)	0.12
Arterial vascularization	134	25 (41%)	36 (49%)	0.33
Venous vascularization	134	37 (61%)	42 (58%)	0.71
Bubble‐like lucency	134	10 (16%)	19 (26%)	0.18
Bronchus sign	134	40 (66%)	43 (59%)	0.43
GGO basal density (HU)	124	−306 (−446, −108)	−298 (−402, −229)	0.34
GGO portal density (HU)	100	−306 (−444, −84)	−257 (−335, −150)	0.59
Lung parenchyma density (HU)	124	−886 (−908, −859)	−882 (−902, −857)	0.41
Difference CT value	92	576 (426, 801)	638 (533, 728)	0.50
Relative attenuation	90	2 (2, 6)	4 (3, 6)	0.025

*Note:* Data are presented as median (interquartile range) of number (%). Comparisons are reported as result of Pearson's chi‐squared test; Wilcoxon rank sum test; Fisher's exact test where appropriate.

Abbreviations: Aci%: acinar percentage, ADK: adenocarcinoma, AIS, adenocarcinoma in situ, ALK: anaplastic lymphoma kinase, EGFR: epithelial growth factor, HU: hounsfield units, Ki67% is the tumor proliferation index, KRAS: rat sarcoma kinase, Lep%: lepidic percentage, LVI: lympho‐vascular invasion, MIA: minimally invasive adenocarcinoma, RATIO is calculated as the ratio between surgical margins and the size in mm of the tumor (pathological report), SCC: squamous cell carcinoma, STAS: spread through air spaces.

### 3.4 Linear Regression and Survival Analysis

Binomial logistic regression was applied to investigate which radiological findings may be considered predictors for consolidation. Adjusted for collinearity data, the model showed with an AIC (Akaike Information Criterion) of 128 that radiological pleural contact and the presence of an arterial vessel surrounded by the mGGO were significant predictors for consolidation (*p* = 0.033 and *p* = 0.028 respectively). The pleural contact seemed to be associated with mGGO showing a lower tendency toward consolidation while the arterial vascular feeding was associated with the development of a solid component (Table 6).

**TABLE 6:** Logistic regression for consolidation of initial pure GGOs.

Predictors	*Z*	Odds ratio	95% CI	*p*
Spiculated	0.739	—	—	0.460
Pleural contact	−2.136	0.084	0.010–0.800	0.033
HU at the initial evaluation	0.736	—	—	0.461
Bronchus sign	0.304	—	—	0.761
Bubble‐like lucency	0.037	—	—	0.971
Vascularized GGO (arterial)	2.191	3.217	1.130–9.140	0.028
Lung density at CT (Hu)	0.450	—	—	0.652
Relative attenuation	−0.841	—	—	0.401

*Note:* Data is presented with the odd ratio only for predictors.

Abbreviations: CT: computed tomography, GGO: ground glass opacity, HU: hounsfield units.

**FIGURE 3:**
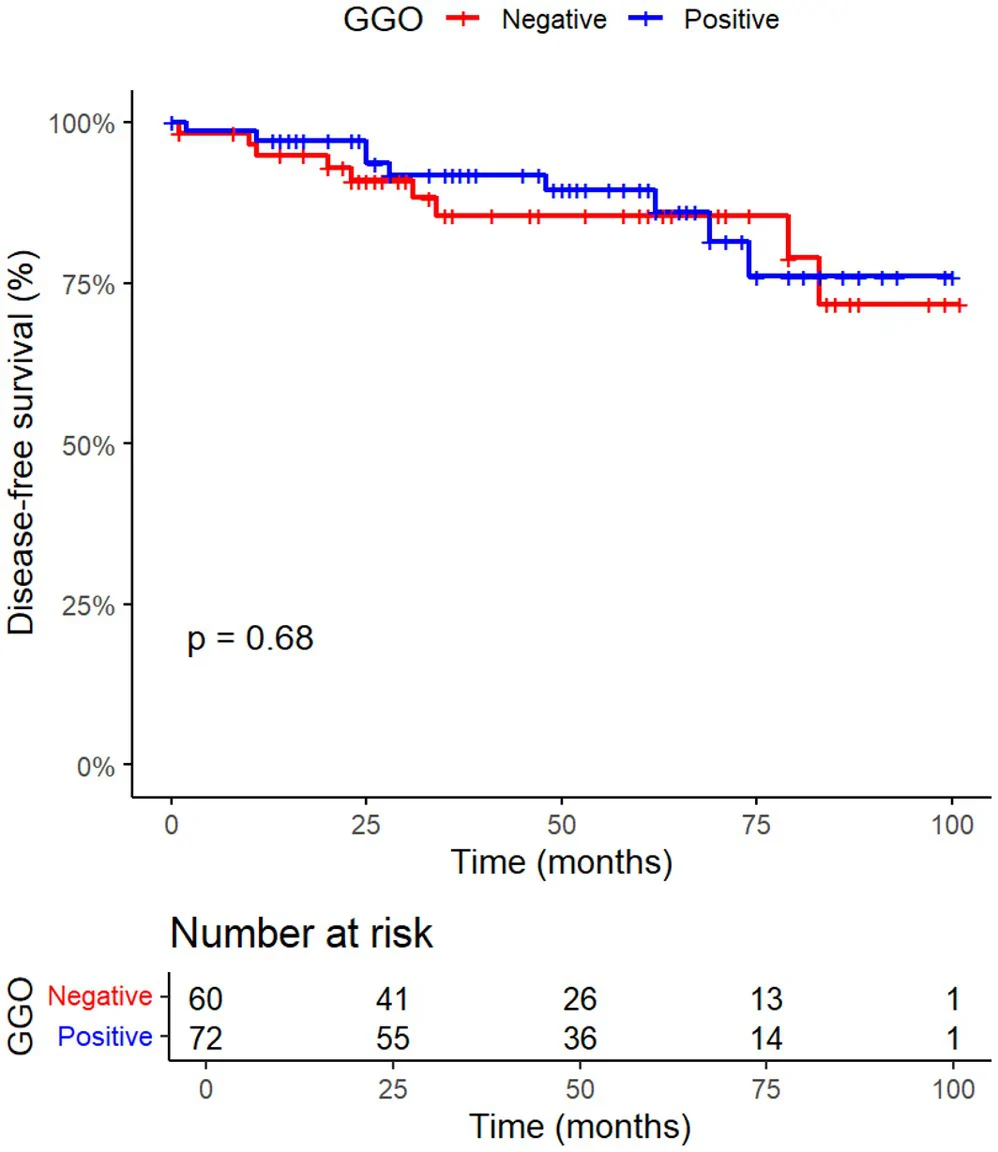
Kaplan–Meier estimates survival according to the GGO feature.

**FIGURE 4:**
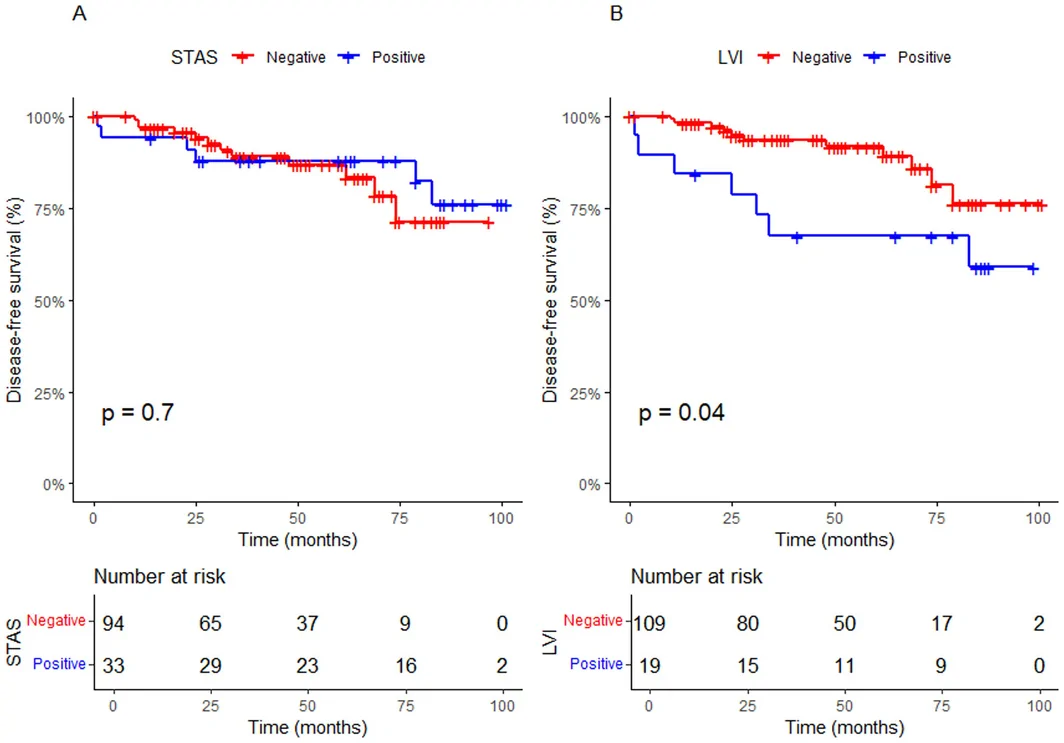
Kaplan–Meier estimates survival according to the presence of STAS (A) and LVI (B).

According to the Kaplan–Meier method, survival of patients with maintained pure GGOs and those who presented at surgery with a consolidated GGO was estimated. 1‐, 3‐, and 5‐year survival rates for pure GGOs were 97.2%, 91.8%, and 81.7%, respectively. In contrast, for patients with consolidated GGO, the corresponding values were 94.9%, 85.5%, and 85.5%, respectively (Log‐Rank *p* = 0.68; Figure 3). Therefore, after checking that no violation of assumptions occurred (*p* = 0.12 for LVI and *p* = 0.34 for STAS), lower survival expectancy was reported in mGGOs presenting LVI at the pathological examination (HR 2.68; 95% CI 1.01–7.10; *p* = 0.047; Figure 4B), while STAS was not significantly associated with survival (HR 1.22; 95% CI 0.29–2.82; *p* = 0.701; Figure 4A).

## 4 Discussion

GGOs may represent initial stages of lung cancer developing as minimally invasive and lepidic features, usually progressing to a more solid form during surveillance. A recent meta‐analysis showed that pure GGOs < 30 mm have a not‐negligible risk of being invasive malignancies [11] eliciting doubts about the conventional “probably benign” behavior of small GGOs nodules [12].

In our study, 61 patients (46%) had pure mGGO which transformed into a semi‐solid lesion, indicating that approximately half of our cohort maintained a stable and pure radiologic feature until surgical resection, even being malignant at pathological analysis. This is in line with previous evidence demonstrating that the absence of consolidation should not be considered a universal benign criterion [13].

Despite all cases were managed with a systematic nodal dissection, we registered only two nodal involvements (1.5%, one N1 and one N2, respectively) among 135 mGGOs. This limited nodal spread confirms larger cohorts where nodal progression in ground glass opacities was anecdotal. Some Authors question that nodal systematic dissection should be superseded in this setting [14, 15], hypothesizing that a nodal sampling may be sufficient and that the accuracy of parenchymal resection matters over deep nodal harvesting, thereby limiting the risk of dissection‐related complications (chylous leakages, esophageal damages, nervous injuries or subcarinal blood loss). Otherwise, tumor relapse occurred in 10 cases (7%), suggesting that routes of tumor spread other than lymphatic drainage may be involved, even at this very early stage, thus warranting further investigation. For example, in our study LVI and STAS were found in 15% and 25% of cases, respectively, indicating that vascular and alveolar spread may be early path of tumor diffusion, driving invasiveness regardless of the tumor consolidation. In particular, extensive STAS, which is frequently found in highly aggressive or advanced NSCLCs, was anecdotical in our study, while limited STAS was mostly represented. Meanwhile, comparative analysis showed a 10% higher quote of LVI in consolidated mGGOs (20% vs. 10%, *p* = 0.11) and arterial vascularization maintained a significant role for consolidation into logistic regression (*p* = 0.028). In addition, a significantly lower survival expectancy was observed in the whole sample in those cases presenting LVI, irrespective of consolidation. Together, these findings are aligned with previous reports showing that mGGOs may benefit from a rich blood supply, which appears to favor tumor consolidation, growth and progression to more invasive malignancies. Liu et al. elegantly demonstrated this relationship using dual‐energy CT scanning [16], highlighting its potential prognostic value in solid early‐stage NSCLCs [17, 18, 19]. According to these findings, the presence of LVI in ground glass opacities should be remarked. Nevertheless, a specific subgroup analysis was impossible due to the small number of cases (only 19 patients with LVI), but the role of vascular supply in pure and semi‐solid GGOs warrants deeper investigations.

Furthermore, the moderate quote of TILs and PD‐L1 expression (43% of cases with moderate or high TILs and 25% of specimens with PD‐L1 ≥ 1%) indicated that immune surveillance is another early response against cancer in mGGOs, although with a lower burden than expected in solid NSCLCs [20, 21]. This hypothesizes that the antigen‐presenting process may occur at an early stage in some cases, indicating the potential for varied immune surveillance mechanisms among patients, even in minimally invasive cancers or in situ settings with malignant potential.

As concerns the protective effect of radiological evidence of pleural contact, the interpretation must be cautious. In fact, pleural contact, that is not retraction but only the presence of GGOs feature at the layer of the visceral pleura, is intriguing. The main interpretation of this feature should be that peripheral pure GGOs may correspond to atypical adenomatous hyperplasia (AAH) and marginally to adenocarcinoma in situ (AIS). They have been reported to be stable over time, with no solid component, with excellent prognosis and in the case of AIS, and with no nodal involvement. Therefore, this result should be plausible, but further confirmations are needed [22]. In the meantime, their position close to the pleural visceral layer might have been cause for early referral compared to other cases of more centrally located mGGOs, driving early diagnostic investigations due to their peripheral position and easy target for a trans‐thoracic needle biopsy.

As concerns the molecular profile, mGGOs showed 47 cases (almost 35%) of EGFR mutated specimens, providing preliminary evidence that molecular pathway disruption is another early feature, aligning with a previous larger Asian study conducted by Tang and colleagues [23].

By a clinical point of view, regardless of consolidation, overall survival trend within 2 years was excellent and proportional (*p* = 0.68), confirming previous experiences reporting good survival expectancies after surgery in this setting alongside negligible risk of nodal spreading in such staging [24, 25, 26].

The evidence that tumor microenvironmental features (LVI and STAS) are precocious findings in pathological mGGOs specimens presents as a significant challenge. As we reported in this study, microscopic evidence of tumor‐associated features appears at a very early stage, and a clear understanding of how these factors progressively invade the peritumoral space has yet to be established.

These findings underscore the considerable radiological and surgical challenges associated with the management of GGOs. Furthermore, the increasing detection of multiple GGOs through lung cancer screening programs represents another emerging issue for thoracic surgeons [27].

Despite our results, some limitations affected our study, and they should be taken into account. First, the single center retrospective study design has limited robustness. In the meantime, the presence of a possible management‐related selection bias between pure and semi‐solid mGGOs should be addressed, even considering that time was not an outcome of our study. Secondly, we excluded wedge resections to include only anatomical resections, but this decreased the overall sample size, affecting the statistical significance and the power of the study. Therefore, our results should find further confirmations in wider cohorts.

## 5 Conclusions

In our study, mGGOs appeared to develop early pathological features, such as STAS and LVI. Since recurrence has been reported even after resection of mGGOs, mechanisms other than the rare occurrence of nodal involvement are likely responsible for postoperative cancer spread. Pleural contact was associated with the persistence of malignant pure GGOs, whereas vascularized GGOs tended to progress toward consolidation. Overall, the lack of a clear consensus regarding the optimal management of mGGOs and the timing of surgical resection suggests that neither active surveillance nor surgical resection should be regarded as universally applicable. Rather, the choice between these strategies should be individualized according to each patient's clinical and radiological characteristics, reflecting the need for a precision medicine approach even at this early stage of disease.

## Author Contributions

**Sara Cecchinato:** validation, visualization, formal analysis. **Alessandro Bonis:** conceptualization, investigation, formal analysis, writing – original draft. **Anna Michielin:** formal analysis, data curation, methodology, software. **Vincenzo Verzeletti:** writing – original draft, visualization. **Chiara Giraudo:** writing – review and editing. **Serena Zanardo:** investigation. **Luigi Lione:** investigation. **Eleonora Faccioli:** project administration. **Giulia Pagliarini:** conceptualization, methodology, data curation, investigation. **Giovanni Maria Comacchio:** data curation. **Marco Mammana:** funding acquisition. **Marco Schiavon:** validation, writing – review and editing. **Giorgio Cannone:** visualization. **Alessandro Rebusso:** software, resources. **Marco De Silvestro:** data curation. **Andrea Dell'Amore:** supervision, writing – review and editing.

## Funding

The authors have nothing to report.

## Conflicts of Interest

The authors declare no conflicts of interest.

## Data Availability

The data that support the findings of this study are available on request from the corresponding author. The data are not publicly available due to privacy or ethical restrictions.
